# Interactive Electronic Pegboard for Enhancing Manual Dexterity and Cognitive Abilities: Instrument Usability Study

**DOI:** 10.2196/56357

**Published:** 2024-06-21

**Authors:** Shih-Ying Chien, Alice MK Wong, Ching-Yi Wu, Sara L Beckman

**Affiliations:** 1 Department of Industrial Design Chang Gung University Taoyuan Taiwan; 2 Department of Physical Medicine and Rehabilitation Chang Gung Medical Foundation Taoyuan Taiwan; 3 College of Medicine Chang Gung University Taoyuan Taiwan; 4 Healthy Aging Research Center Chang Gung University Taoyuan Taiwan; 5 Haas School of Business University of California, Berkeley Berkeley, CA United States

**Keywords:** interactive electronic pegboard, stroke, hand dexterity, cognitive rehabilitation, system

## Abstract

**Background:**

Strokes pose a substantial health burden, impacting 1 in 6 people globally. One-tenth of patients will endure a second, often more severe, stroke within a year. Alarmingly, a younger demographic is being affected due to recent lifestyle changes. As fine motor and cognitive issues arise, patient disability as well as the strain on caregivers and health care resources is exacerbated. Contemporary occupational therapy assesses manual dexterity and cognitive functions through object manipulation and pen-and-paper recordings. However, these assessments are typically isolated, which makes it challenging for therapists to comprehensively evaluate specific patient conditions. Furthermore, the reliance on one-on-one training and assessment approaches on manual documentation is inefficient and prone to transcription errors.

**Objective:**

This study examines the feasibility of using an interactive electronic pegboard for stroke rehabilitation in clinical settings.

**Methods:**

A total of 10 patients with a history of stroke and 10 healthy older individuals were recruited. With a limit of 10 minutes, both groups of participants underwent a series of challenges involving tasks related to manual operation, shape recognition, and color discrimination. All participants underwent the Box and Block Test and the Purdue Pegboard Test to assess manual dexterity, as well as an array of cognitive assessments, including the Trail Making Test and the Mini-Mental Status Examination, which served as a basis to quantify participants’ attention, executive functioning, and cognitive abilities.

**Results:**

The findings validate the potential application of an interactive electronic pegboard for stroke rehabilitation in clinical contexts. Significant statistical differences (*P*<.01) were observed across all assessed variables, including age, Box and Block Test results, Purdue Pegboard Test outcomes, Trail Making Test-A scores, and Mini-Mental Status Examination performance, between patients with a history of stroke and their healthy older counterparts. Functional and task testing, along with questionnaire interviews, revealed that patients with a history of stroke demonstrated prolonged completion times and slightly inferior performance. Nonetheless, most patients perceived the prototype as user-friendly and engaging. Thus, in the context of patient rehabilitation interventions or the evaluation of patient cognition, physical functioning, or manual dexterity assessments, the developed pegboard could potentially serve as a valuable tool for hand function, attention, and cognitive rehabilitation, thereby mitigating the burden on health care professionals.

**Conclusions:**

Health care professionals can use digital electronic pegboards not only as a precise one-on-one training tool but also as a flexible system that can be configured for online or offline, single-player or multiplayer use. Through data analysis, a more informed examination of patients’ cognitive and functional issues can be conducted. Importantly, patient records will be fully retained throughout practices, exercises, or tests, and by leveraging the characteristics of big data, patients can receive the most accurate rehabilitation prescriptions, thereby assisting them in obtaining optimal care.

## Introduction

Worldwide, the aging population continues to increase, with several attendant problems [1,2]. The process of aging entails repercussions that extend beyond mere physiological conditions and encompasses a diverse spectrum of complications [3]. Older individuals are confronted with economic, psychological, and societal predicaments stemming from physical aging [4,5]. Hypertension is a critical condition intricately connected with the occurrence of strokes [6,7]. In 2020, a total of 7.08 million individuals globally died due to cerebrovascular disorders [8]. Encouragingly, continuous advancements in medical technology have increased the survival rate for patients with a history of stroke to 62% [9]. Nonetheless, even in cases of survival, 90% of patients experience residual effects, making rehabilitation approaches pivotal [10,11].

Stemming from damage to cerebral tissue, cerebral stroke gives rise to a variety of distinct neurological symptoms contingent upon the site of injury [12,13]. This often culminates in motor, sensory, and cognitive impairments among patients with a history of stroke, in which reduced attentional focus and memory deficits are common [14,15]. The aftermath of a stroke can have negative effects on patients’ daily lives, occupational status, and social involvement [16,17]. To enhance physical mobility, manual proficiency, and cognitive aptitude, occupational therapy is the gold standard for elevating overall function [18]. Rehabilitation procedures are initiated once a patient’s vital signs stabilize [19]. Clinical evidence indicates that due to significant individual variations among patients with a history of stroke, including age, rehabilitation needs vary [20-22]. Furthermore, older adults predominantly seek to restore ambulatory capacities, whereas younger individuals emphasize intricate fine motor rehabilitation exercises due to occupational demands [23].

In clinical practice, therapists often use calibrated instruments to evaluate and document patients’ manual dexterity and cognitive recovery capabilities in one-on-one settings [24-26] using standard methodologies, such as the Purdue Pegboard Test (PPT) and the Nine-Hole Peg Test [24,27]. However, the use of a countdown timer to measure tasks within specific time frames has been validated as an effective means to infer attention, cognition, and manual dexterity capabilities in clinical contexts [28,29]. However, there remain substantial challenges, including human resource depletion, increased time expenditures, difficulties in effective disease progression tracking, and recording errors. Acharya et al [24] emphasized the inherent delay, particularly in response time, with the traditional interactive training method. Compared with the current setup, there is a consistent observation of higher measured timing. Taking the commercially available Neofect Smart Pegboard as an example, it serves as an electronic pegboard [30]. While it offers several advantages, it does not feature long-term tracking and precise prescriptions for individual patients. Furthermore, using the electronic prototype of the Grooved Pegboard Test proposed by Al-Naami et al [31] in 2021 as an example, its operational efficiency shows no significant differences compared with the traditional method of manually recording rehabilitation outcomes. This experiment validates the feasibility of an electronic pegboard test to measure hand-time dexterity with impaired hand functionality, indicating comparable or even superior effectiveness when compared with the conventional manual recording approach.

In this study, we used electronic sensing techniques integrated with Wi-Fi and tablet devices to achieve a higher level of precision in evaluating tasks and time of completion [32]. This digitized approach facilitates accurate documentation of the intricacies associated with each practice and assessment, thereby enhancing the overall precision of the rehabilitation process [33,34]. The principal objective of this study was to subject the prototype to initial evaluation and testing involving patients with a history of stroke and healthy older individuals. We aimed to determine the appropriateness of the set difficulty levels, time constraints, and speed of the prototype.

## Methods

### Overview

The experimental apparatus consisted of an iPad, 5 color-sensitive building blocks, and 3 variations of task casings. The system’s underlying sensing mechanism relied on the modulation of capacitance values resulting from the interaction between the sensing electrodes of the panel and the human body. The conductive building blocks generated stimulation signals that served as surrogate agents for fingers.

**Figure 1:**
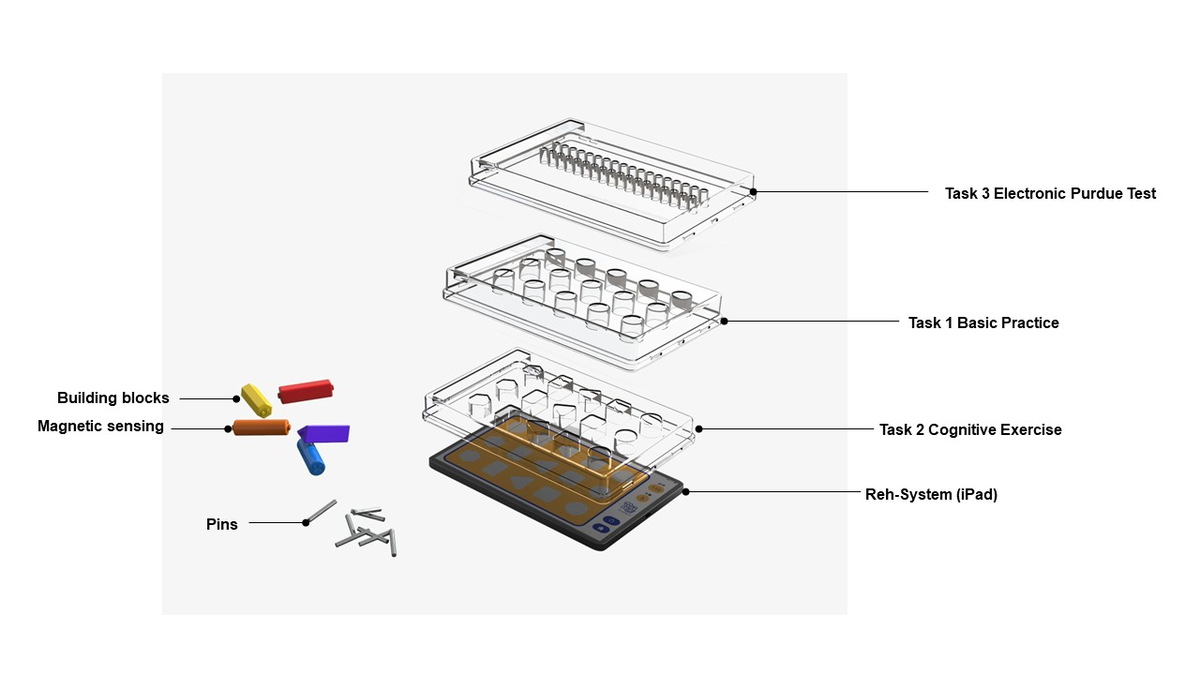
Design of the proposed system.

**Figure 2:**
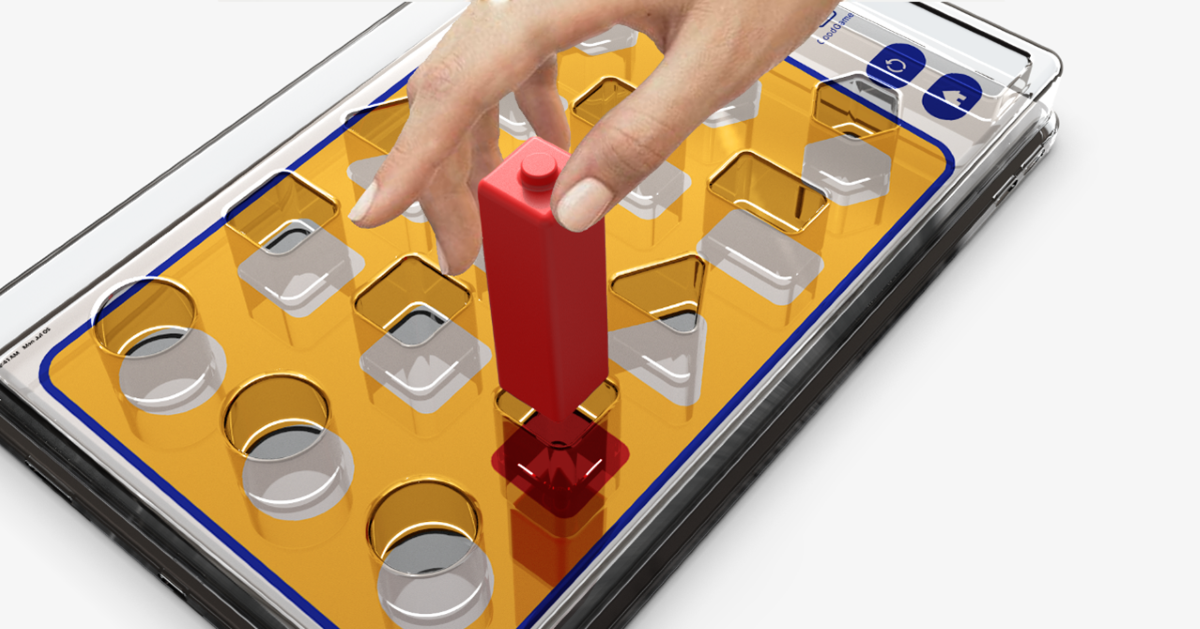
Interactive sensor blocks and tablet interface scenario.

A schematic representation is shown in Figure 1. Paired with the distinctive visual patterns on the back of each building block, these visual patterns upon contact with the iPad screen were detected and recognized through pressure sensing. This design was aimed at assessing the responsiveness, visual acuity, and color perception abilities of the participants during rehabilitation interactions (Figure 2).

All rehabilitation tasks and exercises integrated time calculation and countdown functions. Patients were given the option to choose between “independent practice” and “interactive practice” modes. During independent practice, after the “start” button was pressed and the countdown timer initiated, randomized questions were presented. Each practice session was preconfigured for a duration of 10 minutes, and completion and error rates were captured.

**Figure 3:**
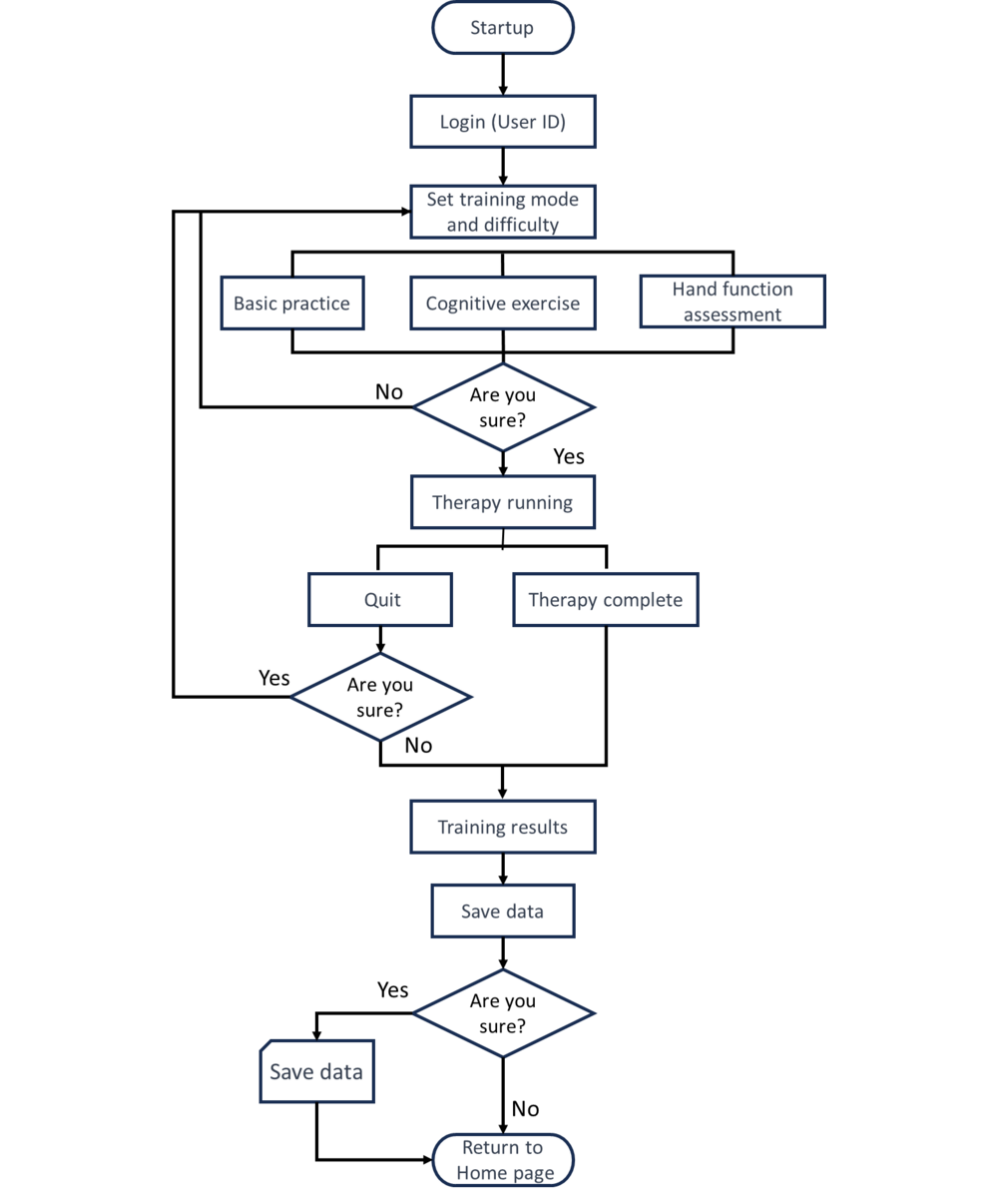
Flowchart of the proposed system operation.

For interactive practice, therapists preset practice durations and modify difficulty levels (rehabilitation prescriptions). Through a Wi-Fi connection, therapists administer questions to make an online assessment of patients’ abilities. After patients perform the tasks, both patients and therapists receive practice and rehabilitation reports, with all exercise records automatically stored in the cloud. A flowchart illustrating the operation of the proposed pegboard is shown in Figure 3.

The proposed system encompasses 3 distinct modes, each presenting varying levels of complexity.

### Basic Practice

**Figure 4:**
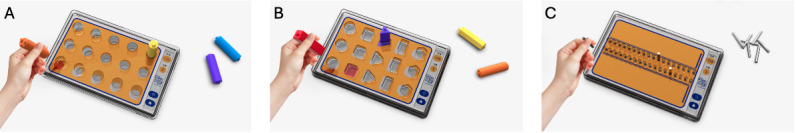
Three modes of the proposed system: (A) basic practice; (B) cognitive exercise; and (C) Electronic Purdue Test.

In basic practice (BP)–1 mode, users are assigned the task of associating the building blocks with the corresponding positions guided by reminder lights on the tablet screen (Figure 4A). Users earn points when they correctly insert the blocks into the panel’s corresponding positions. In the intermediate level (BP-2), users place the blocks in the corresponding positions as indicated by the lights of the screen within the given time frame while concurrently considering the variety of colors presented. The advanced level (BP-3) introduces a speed variable to increase the complexity of the task.

### Cognitive Exercise

In cognitive exercise (CE)-1 mode, patients are required to distinguish the shapes of the building blocks and place them according to the patterns displayed on the screen. Users earn points when the blocks are correctly positioned. Upon advancing to the CE-2 level, users need to not only identify the corresponding shaped blocks but also distinguish the colors indicated by the lights. Points are awarded only when both the shape and the color are correct. In addition, this mode introduces varying levels of complexity related to color discrimination and speed (Figure 4B).

### Electronic Purdue Test

In the design of the electronic Purdue Test (EPT) level, adherence to the principles of the PPT was paramount. The illuminated signals were meticulously crafted to guide patients in sequentially inserting pegs into corresponding holes (Figure 4C). The assessment consists of distinct 30-second trials for the right, left, and both hands, with individual scores recorded and aggregated. In addition, a 60-second bilateral combination test is administered once. This comprehensive set of evaluations is repeated 3 times. The platform automatically calculates the average score, which serves as the test score. Unlike traditional training, the device guides patients to place pegs into corresponding positions through the use of light signals, which remain illuminated until the pegs are properly placed.

Given the inherent variability in individual patient capabilities, prescribed treatments should differ. To facilitate precision health care, the system incorporates a user login mechanism to generate personalized digital rehabilitation plans and records. The proposed design comprises 3 different modes and 3 difficulty levels of exercises. Preestablished exercises encompass directional movements (including upward, downward, leftward, and rightward motions). During the initial stages of the rehabilitation regimen, the system uses a mechanism of stochastic question generation. As the system accumulates practice data, it systematically discerns and assimilates the individual requirements of each patient, thereby tailoring subsequent questions to enhance areas of observed weakness.

### Research Aims

This research had the following aims: (1) we sought to investigate the suitability of the time and difficulty settings for both patients with a history of stroke and healthy users, (2) we explored the correlation between hand function and cognitive abilities, and (3) we conducted a usability questionnaire for the proposed system.

### Participant Recruitment

A total of 20 older adults aged between 65 and 80 years were recruited: 10 (50%) patients with a history of stroke and 10 (50%) individuals with no history of strokes. Prior to their inclusion, all participants provided signed informed consent. All participants exhibited right-handed dominance. The inclusion criteria were delineated based on the following: (1) capacity to independently maintain a seated position for a duration exceeding 20 minutes, unaided by external assistance; (2) possession of fundamental communication skills; and (3) relatively uncomplicated functional performance in the assessment of daily life activities. As a safeguard against potential trial-related risks, individuals with a history of recurrent stroke, severe muscular atrophy, or pronounced physical frailty were excluded.

### Experimental Procedure

**Figure 5:**
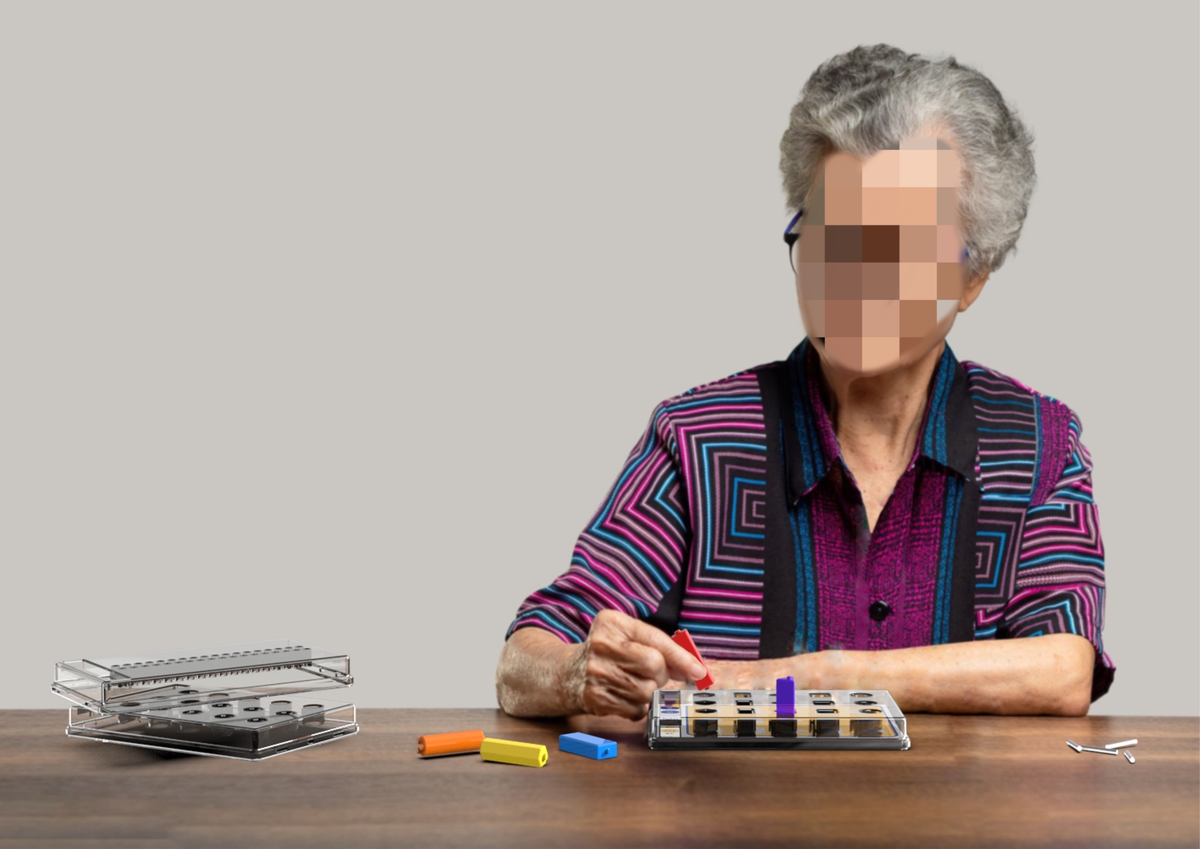
Image of the proposed system in use.

All participants underwent the Box and Block Test (BBT) and the PPT to assess manual dexterity, as well as an array of cognitive assessments, including the Trail Making Test (TMT) and the Mini-Mental Status Examination (MMSE), which served as a basis to quantify participants’ attention, executive functioning, and cognitive abilities. Figure 5 shows an image of the proposed system in use.

The experimental session was conducted on a one-on-one basis. The prototype was positioned before each participant, and the 15 pegs, consisting of 5 distinctive colors integral to the interactive system, were methodically arranged adjacent to the central apparatus. Participants sequentially underwent 3 testing modes (BP, CE, and EPT), using their right hand exclusively. Except for the EPT, which followed the PPT criteria, the length of the BP and CE tests was 10 minutes each. The test outcomes encompassed the number of correct responses, completion time, and rehabilitation reports, all of which were concurrently displayed, stored within the apparatus, and uploaded to the cloud platform.

To gain a thorough understanding of users’ interactions with the proposed system, we used the System Usability Scale [25] with a 5-point Likert scale to reveal users’ perceptions regarding aspects of system acceptance, design appeal, and perceived task difficulty.

### Statistical Analysis

This study used SPSS software (version 20; IBM Corp) for statistical analyses. Data analysis involved a comparative assessment between patients with a history of stroke and healthy older individuals, exploring both demographic characteristics and scores obtained from the proposed system, with the Wilcoxon rank sum test used for statistical analysis. To elucidate the potential associations between participants’ manual dexterity and cognitive faculties among patients with a history of stroke, this study also used the Spearman rank correlation coefficient, with statistical significance set at *P*<.05.

### Ethical Considerations

A total of 20 participants were recruited for this study. All participants provided informed consent by signing a consent form, and the study was conducted in accordance with institutional review board (IRB) regulations using anonymized data, with personal information removed and replaced by codes. No participants withdrew from the study during the research period. To ensure the validity and fairness of the experiment, no monetary or material benefits will be provided during the trial period, in accordance with the IRB application statement. Participants are expected to provide genuine feedback on the product developed in this project based on their intuitive reactions.

The research was conducted within the Department of Physical Medicine and Rehabilitation at Chang Gung Hospital, Taiwan and received approval from the Research Ethics Committee for Human Subject Protection of Chang Gung Medical Foundation (IRB: 202301197A3).

## Results

This study included a total of 20 participants (10 patients with a history of stroke and 10 healthy participants). With a limit of 10 minutes, both groups of participants underwent a series of challenges involving tasks related to manual operation, shape recognition, and color discrimination. The statistical analysis revealed statistically significant discrepancies between patients with a history of stroke and healthy participants across all variables (*P*<.05). These differences were evident in all assessed parameters, indicating the potential of the equipment to serve as an assessment tool for both motor and cognitive abilities in both healthy individuals and patients with a history of stroke, with additional training and testing capabilities (Table S1 in Multimedia Appendix 1).

Among older participants who had not experienced a stroke, performance in tasks involving the dominant hand (right hand) during the BBT and the PPT as well as cognitive performance in the TMT was notably superior to those who had experienced a stroke (Figure S1A and S1B in Multimedia Appendix 1). However, no significant differences were observed between the 2 groups in the MMSE test, which assessed memory abilities (Table S2 in Multimedia Appendix 1). Furthermore, in the MMSE test assessing memory abilities, both groups of subjects showed significant differences (*P*<.01) (Table S2 in Multimedia Appendix 1).

Statistical analysis revealed significant negative correlations between performance in the BP-1 or CE-1 task and dexterity tests (*P*<.01). In addition, there were significant correlations between multicolors (BP-2 or CE-2) and dexterity or cognitive tests and a significant negative correlation between scores in the EPT and cognitive performance on the TMT-A and MMSE tests (*P*<.05) (Table S3 in Multimedia Appendix 1). The number of correct answers was used as the score in BP and CE; the time required was used as the score in BP, CE, and EPT in the single and multicolor tests. In terms of usability, 40% (4/10) of patients with a history of stroke and 60% (6/10) of healthy participants deemed the prototype user-friendly (Figure S2A in Multimedia Appendix 1).

Furthermore, all healthy individuals (100%) and majority of patients with a history of stroke (90%, 9/10) found the proposed system highly engaging. During the more demanding CE training, more than 80% (8/10) and 50% (5/10) of healthy participants and patients with a history of stroke, respectively, considered the system both challenging and stimulating. Participants additionally expressed a positive disposition toward the EPT and provided overall positive feedback (Figure S2B in Multimedia Appendix 1).

In assessing task difficulty, nearly 80% (8/10) and 60% (6/10) of healthy participants and patients with a history of stroke, respectively, perceived the BP training tasks as easy and straightforward. However, as participants advanced to the more challenging CT training, there was a noticeable increase in the perceived complexity of tasks, in which only 40% (4/10) and 20% (2/10) of healthy participants and patients with a history of stroke, respectively, found this phase easy. Moreover, 60% (6/10) of healthy participants found the EPT straightforward, while 40% (4/10) of patients with a history of stroke indicated a moderate level of challenge associated with the EPT training (Figure S2C in Multimedia Appendix 1).

## Discussion

### Principal Findings

Across the 5 tasks investigated in this study, the stroke rehabilitation group exhibited significantly lower scores in the use of the proposed system compared to the healthy participants. In accordance with previous research, advancing age and disease manifest changes and declines in hand function, muscle strength, agility, and cognitive abilities [35,36]. This was evident in the use of the proposed system.

Previous studies have highlighted the repercussions of cerebral damage on patients with a history of stroke, such as compromised cognitive, motor, sensory, and functional capabilities, as well as pain, balance issues, visual challenges, and restricted engagement in activities [37,38]. Sudden cognitive deterioration occurs as a result of these conditions, with 5% of patients with a history of stroke exhibiting dementia symptoms [39,40]. Thus, the augmentation of hand function rehabilitation for patients is imperative. Our findings underscore a close interrelation between manual dexterity and cognitive aptitude [41]. In future research, we intend to explore the use of the proposed system paired with auditory and visual cues to ascertain its potential to guide and enhance visual acuity, attention, and cognitive capabilities among cohorts of different ages and individuals afflicted with cerebral impairments.

The BP and CE tests encompassed factors of both single-color and multicolor conditions. Participants encountered operating difficulties as cognitive demands increased. The CE assessment included recognition of color and object shape, where performance consistently declined across all participants. This observation aligns with previous findings indicating a decline in change detection accuracy with an increase in cognitive load [26,27]. Consequently, as the number of colors increased, the attentional burden on the patients correspondingly increased. This suggests that a graded system with different levels of difficulty might be useful.

During the progression of single-color BP-1 and CE-1 sessions, negative correlations were observed in dexterity tests. In the context of multicolor BP and CE sessions, a distinct and significant correlation was found between the use of multicolors and performance levels on the TMT-A test. The test results suggest that when participants engaged in color recognition and discrimination tasks, the attentional demands for single-color and multicolor tasks differed. In other words, multicolor exercises presented an increased cognitive challenge, affecting manual dexterity and attention switching.

The majority of participants found the proposed system highly user-friendly, in part because the size of the system resembled traditional training pegboards, which maintained familiarity and reduced the need for adaptation. As therapists were not required to manually record participants’ actions or time them with stopwatches, this design was advantageous for both users and evaluators. In the various tasks, approximately 60% (12/20) to 70% (14/20) of all participants found BP training to be interesting, while 40% (8/20) to 60% (12/20) of the participants considered the EPT tasks engaging. Regarding the difficulty level, 60% (12/20) to 70% (14/20) of the participants perceived BP training as relatively easy; however, as operating constraints increased (such as color and shape elements), the participants commonly reported that tasks became more challenging and demanding. This finding is consistent with prior research indicating that increasing the difficulty to match users’ current abilities enhanced their confidence, maintained attention and engagement in tasks, and promoted a more positive and enjoyable acceptance of new challenges. In this preliminary experiment, neither patients with a history of stroke nor healthy participants were able to complete the tasks within the allotted time, and none of the participants achieved a perfect score. This is likely attributable to the time constraints imposed by the experimental design or the capabilities of the users. Therefore, future studies will include basing task difficulty settings on user performance, similar to leveling up in a video game. The gamification of rehabilitation, in addition to fostering effective interactivity, is facilitated by the incorporation of voice and music assistance. This approach contributes to enhancing the enjoyment of rehabilitation, transforming it from a tedious and uninteresting process. Furthermore, it effectively redirects patients’ attention away from pain, thereby augmenting the overall appeal of the rehabilitation process.

The proposed system is equipped for practice, training, and assessment. The majority of similar products on the market predominantly focus on training manual dexterity and do not offer timing and recording functions [28-30]. Certain designs acknowledge the significance of cognitive training and use shape as a cognitive judgment criterion; however, these designs lack elements that enhance the attention of rehabilitation patients, such as auditory cues, visual stimuli, or color-guided prompts. This deficiency in interactive mechanisms often results in users struggling to sustain or commit to rehabilitation efforts. Finally, in terms of assessment functionality, contemporary clinical practice still relies on manual documentation and human intervention for upper limb assessments. The proposed system not only incorporates timing and counting features during upper limb assessments but also introduces guiding and competitive elements, positioning patients to achieve better recovery outcomes. Currently, the system is converting data from each patient’s rehabilitation sessions into charts. This aids both patients and health care professionals in gaining a more comprehensive understanding of the rehabilitation and recovery status. This system is thus highly advantageous compared with current commercial products [32-34,42]. The interactive electronic pegboard integrates the merits of existing market offerings and further introduces automated assessment and scoring mechanisms for accurately placed pegs. By surpassing the limitations inherent in conventional fixed training paradigms, this system systematically and comprehensively records the training progress of each case. As data accumulate via a learning model, the system develops a profound understanding of user-specific requirements and consequently extrapolates optimal and customized training regimens tailored to individual users, representing a major step toward precise rehabilitation goals. Finally, the proposed system exhibits greater versatility in its training curriculum and offers increased variability and flexibility. Rehabilitation with digital tools will no doubt significantly enhance users’ interest and attention.

Our preliminary investigation indicates that the proposed system is beneficial in the training, assessment, and testing of patients with a history of stroke. The outcomes showcase positive responses concerning hand function training and cognitive ability assessment among patients with a history of stroke. However, to ascertain reliability and validity, a greater number of participants, including diverse age groups and individuals with cerebral impairments, should be recruited in future investigations [35].

Patients with a history of stroke often grapple with diminished motivation for rehabilitation and a lack of immediate feedback, hindering their ability to maintain consistent participation in rehabilitation regimens [36-38]. While the platform devised in this study uses auditory and visual cues to encourage perseverance in rehabilitation, there remains room for improvement in configuring different challenge levels and real-time feedback mechanisms based on varying patient capacities [39-41]. This is a pivotal objective for refinement and enhancement.

### Conclusions

The primary objective of this research was to bridge the gap between clinical requirements and product development through customized rehabilitation training based on individual differences. Through the analysis and assessment of data and providing personalized training modes tailored to specific differences, we aimed to predict patients’ hand dexterity and cognitive functional abilities. We thus developed the interactive electronic pegboard, a novel software- and hardware-integrated system for stroke rehabilitation, with the purpose of evaluating dexterity and cognitive functions through various task types and multidemonstration patterns. Preliminary findings indicate the efficacy of the system for training and assessment. The ultimate goal of this research is to develop an intelligent system capable of delivering individualized optimized rehabilitation regimens based on the varying needs of users.

## Appendix

Multimedia Appendix 1 Detailed participant data and analyses.

## Abbreviations

BBT: Box and Block Test
BP: basic practice
CE: cognitive exercise
EPT: Electronic Purdue Test
MMSE: Mini-Mental Status Examination
PPT: Purdue Pegboard Test
TMT: Trail Making Test

## Data Availability

The data sets generated and analyzed during this study are available from the corresponding author on reasonable request.
