# Supplementary material for: Interactive Electronic Pegboard for Enhancing Manual Dexterity and Cognitive Abilities: Instrument Usability Study
Source: JMIR Hum Factors. 2024 Jun 21;11:e56357. doi: 10.2196/56357 (PMC11226928; doi:10.2196/56357)
Supplement: Multimedia Appendix 1 [file humanfactors_v11i1e56357_app1.docx]

**Table S1.** The *t*-test comparison between the stroke and healthy groups.

| **Variables** | **Stroke patients**  **(*n* = 10)** | **Healthy participants**  **(*n* = 10)** | ***P* value** |
| --- | --- | --- | --- |
| Age | 75.8±8.1 | 75.6±8.5 | 0.96 |
| BBT score | 55.1±5.7 | 67±3.7 | <0.01* |
| PPT score | 13.3±2.9 | 16.2±2 | 0.02* |
| TMT-A time | 61.3±10.3 | 47.2±9.9 | 0.01* |
| MMSE | 22.6±4.2 | 27.3±1.8 | <0.01* |
| One colored-BP  Number of correct answer | 8.7±2.4 | 12.3±1.6 | <0.01* |
| Multiple colors-BP  Number of correct answer | 6.9±1.6 | 11.1±1.7 | <0.01* |
| One colored-CE  Number of correct answer | 7.5±2 | 10.8±1.6 | <0.01* |
| Multiple colors-CE  Number of correct answer | 6±1.6 | 10.1±1.9 | <0.01* |
| One colored-EPT  Number of correct answer | 13.8±2.3 | 21.1±7.1 | 0.01* |
| One colored-BP  Completion time | 66.9±13.5 | 46.4±9.5 | <0.01* |
| Multiple colors-BP  Completion time | 129.1±11.9 | 76±5.1 | <0.01* |
| One colored-CE  Completion time | 80.5±9.4 | 65.9±10.2 | <0.01* |
| Multiple colors-CE  Completion time | 160.1±15.1 | 122.2±13.8 | <0.01* |
| One colored-EPT  Completion time | 98.8±28 | 74.9±9.6 | 0.02* |

1. Mean ± standard deviation
2. *p<0.05

**Table S2.** Demographic data of participants.

| Participants | Stroke patients  (*n* = 10) | Healthy participants  (*n* = 10) | *P* value |
| --- | --- | --- | --- |
| Age (years) | 75.8±7.7 | 75.6±8.1 | ** |
| Gender (male/female) | 6/4 | 5/5 | - |
| BBT score | 55.1±5.4 | 67.0±0.7 | * |
| PPT score | 13.3±2.8 | 16.2±1.9 | ** |
| TMT-A | 61.3±9.7 | 47.2±9.4 | ** |
| MMSE | 22.6±4.0 | 27.3±1.7 | ** |

BBT: Box and Block Test; PPT: Purdue Pegboard Test; TMT: Trail Making Test; MMSE: Mini-Mental State Examination.

**Table S3.** Correlations between proposed system scores and dexterity/cognitive evaluations.

| Evaluations | BBT | PPT | TMT-A | MMSE |
| --- | --- | --- | --- | --- |
| BP-1 | -0.63^**^ | -0.50^**^ | 0.21 | 0.32 |
| BP-2 | -0.58^**^ | -0.68^**^ | 0.41^*^ | 0.52^*^ |
| CE-1 | -0.72^**^ | -0.44^**^ | 0.29 | 0.37 |
| CE-2 | -0.41 | -0.42^*^ | 0.53^**^ | 0.61^**^ |
| EPT | 0.34 | 0.41 | -0.50 | -0.55^*^ |

Spearman’s rank correlation analysis (n=20), *: p<0.05, **: p<0.01

BP: Basic Practice; CE: Cognitive Exercise; EPT: Electronic Purdue Test; BBT: Box and Block Test; PPT: Purdue Pegboard Test; TMT: Trail Making Test

1.
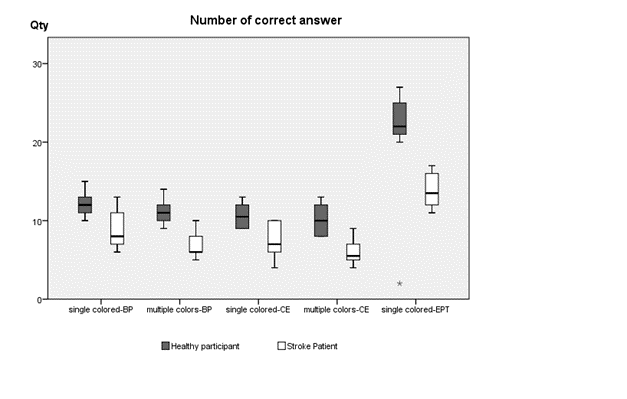
Number of correct answers
2.
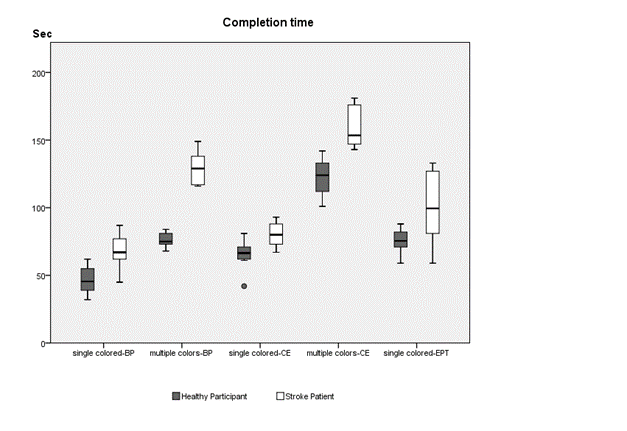
Completion time

**Figure S1.** User performance on proposed system.


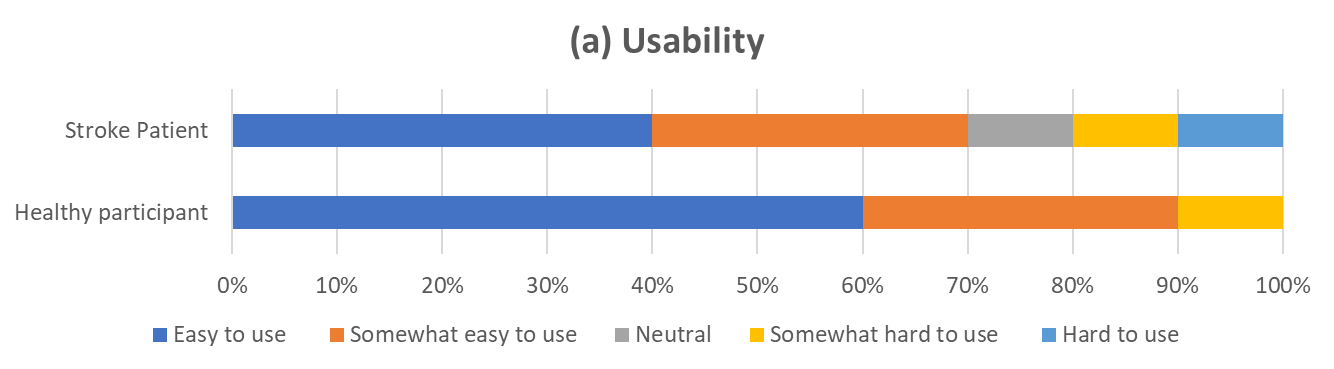

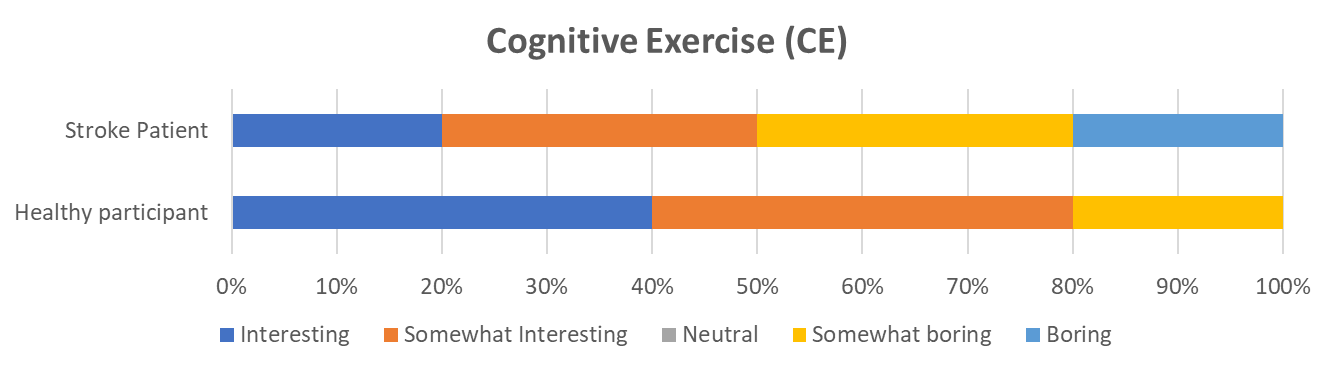

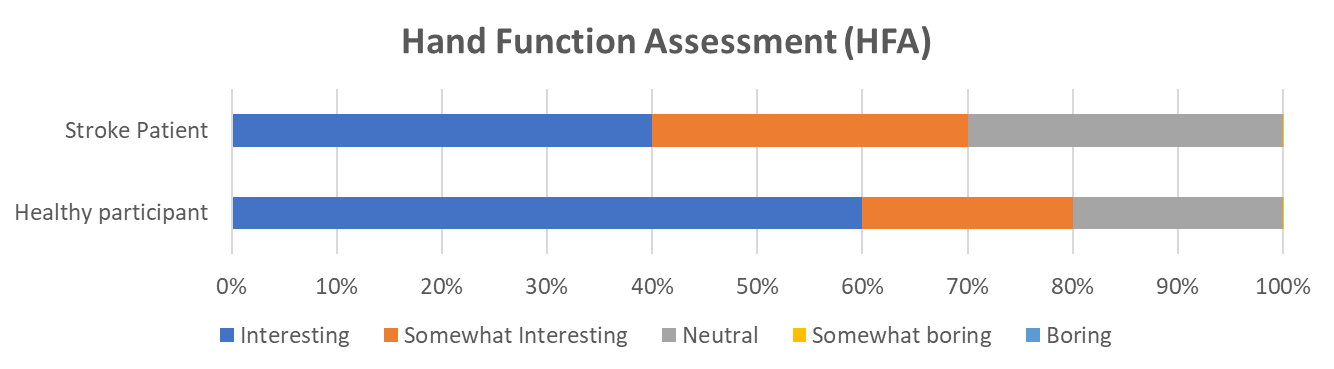

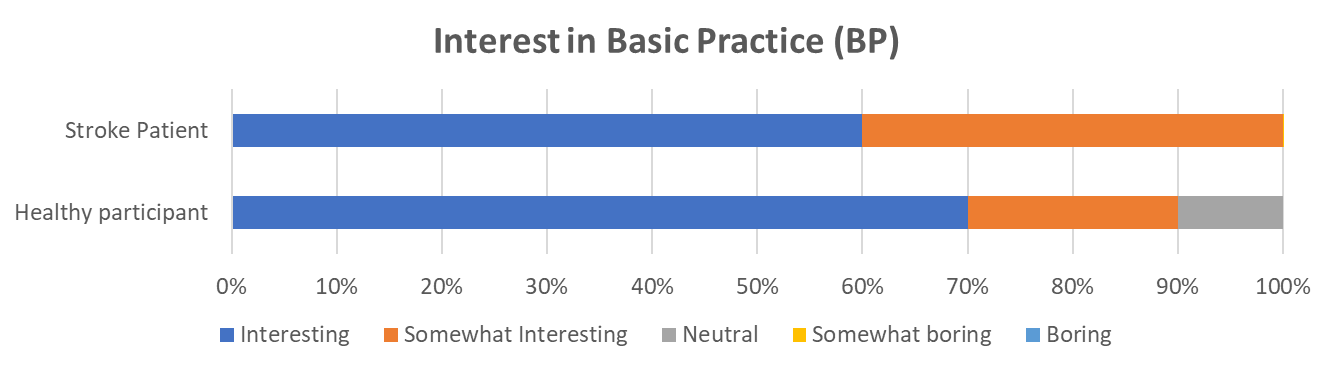


**(b) Interest**

**(C) Difficulty**


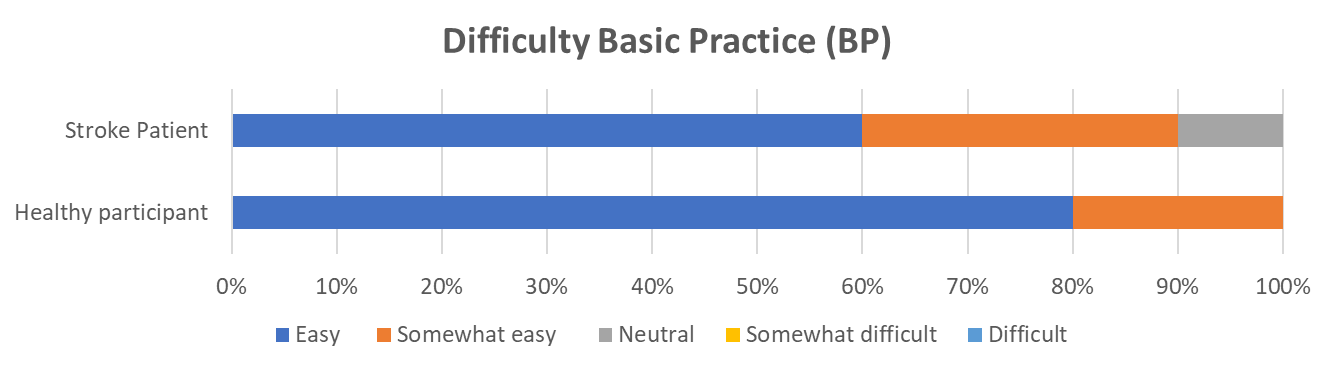


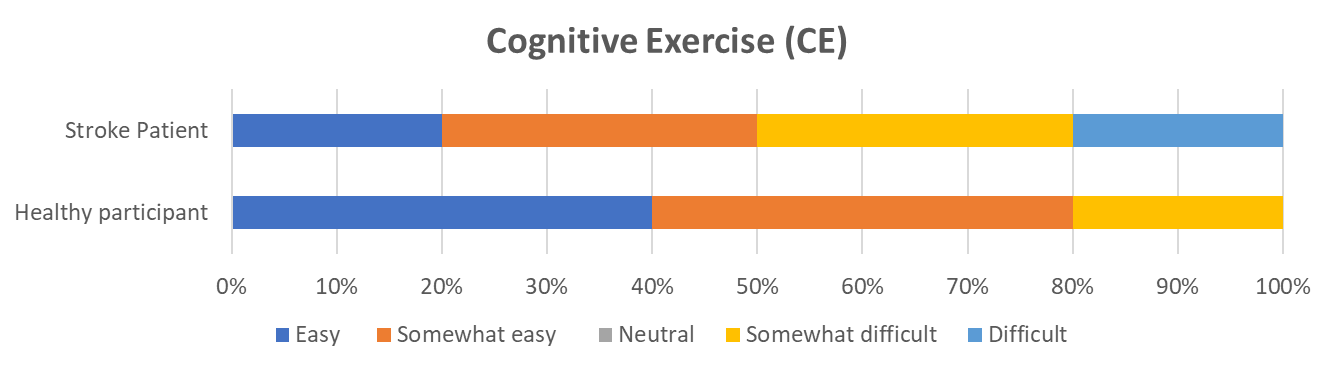


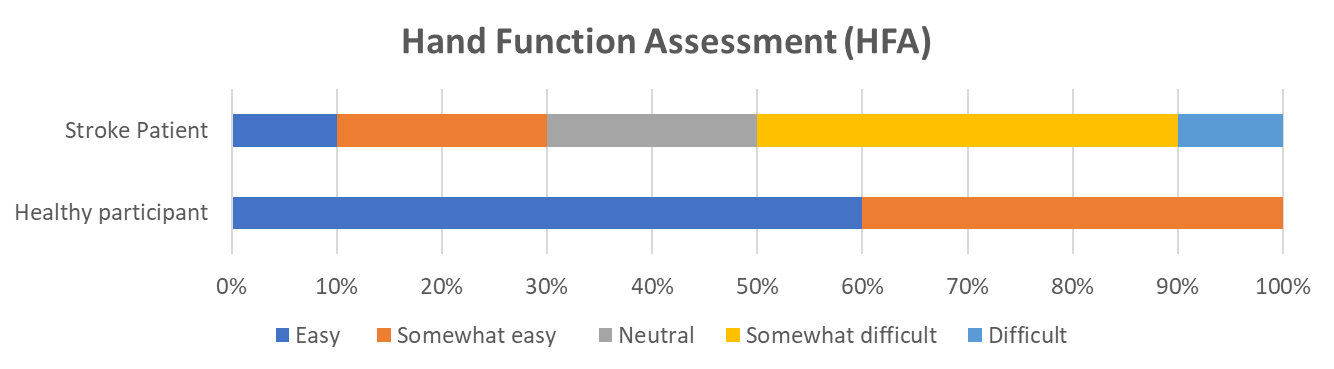


**Figure S2.** Subjective evaluations of proposed system: stroke patients (n=10), healthy participants (n=10).
